# Senescence-driven osteonecrosis of the femoral head in the elderly—a distinct pathophysiological entity

**DOI:** 10.3389/fmed.2025.1653037

**Published:** 2025-12-10

**Authors:** Tong-jie Yang, Tian-xin Chen, Yu Zhang, Ye Luo, Peng-peng Wen

**Affiliations:** 1 Wangjing Hospital, China Academy of Chinese Medical Sciences, Beijing, China; 2 Zhongshan Hospital, Xiamen University, Xiamen, Fujian, China; 3 First Affiliated Hospital of Wenzhou Medical University, Wenzhou, Zhejiang, China

**Keywords:** osteonecrosis, femoral head, aging, senescence, subchondral insufficiency fracture, bone marrow adiposity, angiogenesis, inflammaging

## Abstract

This article conceptualizes “Senescence-Driven Osteonecrosis of the Femoral Head” in elderly patients as a distinct pathophysiological entity, differing from classic osteonecrosis of the femoral head (ONFH) by its primary age-related etiological factors. It hypothesizes that this condition arises from a convergence of vascular fragility, impaired bone mechanoadaptation, and systemic inflammaging. Epidemiological patterns reveal under-recognition and diagnostic delays, with presentations often mimicking osteoarthritis. The manuscript examines endothelial cell senescence, microvascular dysfunction, increased bone marrow adiposity—reducing perfusion and osteogenic capacity—and chronic inflammation in the aging femoral head. Emerging omics and biomechanical evidence, including subchondral insufficiency fractures, highlight molecular and structural differences contributing to collapse. Conventional joint-preserving treatments often fail in older individuals, prompting exploration of novel therapies like senolytics, vasculoprotective agents, and early mechanical support. This work advocates for a new framework integrating geriatric comorbidities and senescence biology for tailored diagnosis, prevention, and treatment of this debilitating condition.

## Introduction

Osteonecrosis of the femoral head (ONFH) typically affects young to middle-aged adults, often linked to risk factors like high-dose corticosteroids, excessive alcohol, or trauma (1). It commonly impacts those in their 30s and 40s (2), being a leading cause for hip arthroplasty in individuals under 50 (3). In contrast, idiopathic ONFH in the elderly (late 60s+) has been considered relatively uncommon, historically often misattributed to secondary causes or osteoarthritis (4). However, emerging evidence suggests aging itself confers a unique susceptibility to ONFH (5–9), independent of classic triggers, through mechanisms involving senescence, vascular aging, and osteoporosis, potentially forming a distinct pathophysiological entity. This article hypothesizes that ONFH in the elderly is driven by a convergence of senescence-related processes—microvascular endothelial aging, marrow adiposity, impaired osteogenic repair, and “inflammaging”—collectively predisposing the femoral head to ischemia, subchondral fracture, and collapse, even without classic risk factors.

In younger patients, ONFH typically requires a precipitating insult (e.g., corticosteroid-induced lipid deposition or an acute vessel injury) to disrupt the delicate blood supply of the femoral head (1). In older patients, we posit that the baseline state of the femoral head is one of diminished “physiologic reserve”: the small end-arteries of the femoral head may already be narrowed by arteriosclerosis or endothelial senescence, the bone remodeling capacity is blunted, and the marrow environment is skewed toward adipogenesis at the expense of osteogenesis. Under these conditions, even minor stresses or subclinical injuries could tip the balance and initiate an ischemic or structural collapse of subchondral bone.

To substantiate “senescence-driven” ONFH, this article systematically explores its clinical/radiographic patterns, molecular features, and management challenges. We examine overlooked epidemiology and diagnostic hurdles in older adults, then analyze key systemic aging factors (endothelial senescence, altered marrow perfusion/composition, inflammaging) and comorbidity exacerbation. The article contrasts imaging phenotypes and molecular signatures (senescence markers, apoptotic profiles) between elderly and younger ONFH patients to highlight aging-driven pathogenesis. We also analyze biomechanical deterioration of the aging femoral head, particularly subchondral bone vulnerability. Finally, we illuminate therapeutic implications, explaining standard intervention failures in older patients and proposing age-tailored strategies (senolytics, vasculoprotective agents, early biomechanical support).

By redefining elderly ONFH as a geriatric syndrome of bone fragility and microvascular insufficiency, this article stimulates new research and clinical frameworks. We integrate radiological, molecular, biomechanical, and clinical data into a cohesive senescence-driven osteonecrosis model, potentially improving diagnostic vigilance, preventive strategies, and personalized elderly ONFH treatments.

## Epidemiology and diagnostic challenges in elderly ONFH

The true prevalence of ONFH in the elderly is likely underestimated, primarily due to “diagnostic overshadowing” by the far more common osteoarthritis (OA) (10). Consequently, hip pain in patients over 60 is often initially misattributed to OA or vertebral radiculopathy, leading to diagnostic delays (11). Consequently, elderly patients often present with advanced ONFH, characterized by femoral head collapse and joint space narrowing, findings that can mimic severe OA on plain radiographs (11). Supporting this, Shibayama’s research on idiopathic ONFH in individuals over 60 revealed a pattern of rapid femoral head collapse (4). A key distinction in these older patients was the frequent absence of the sclerotic “demarcation line” typically seen around necrotic lesions in younger individuals. Instead, they often exhibited widespread bone destruction, sometimes extending into previously healthy bone (4). This rapid progression and the lack of a clear reparative boundary suggest that ONFH in the elderly may manifest as a more aggressive and diffuse disease process, potentially linked to an inadequate healing response in aging bone.

These diagnostic delays are critical. Elderly patients with early ONFH may report insidious, intermittent pain; however, without obvious risk factors, the index of suspicion is often low, and initial radiographs can be unrevealing or show only minor changes (12). Advanced imaging, such as MRI, is frequently deferred until pain worsens or collapse ensues, by which time the window for joint-preserving interventions may have passed (13). This contrasts with younger patients, in whom a history of steroid use or hip trauma might prompt an earlier MRI before collapse (14). A high clinical suspicion is therefore needed when evaluating an older patient with unexplained hip pain, especially if pain is acute-onset or out of proportion to radiographic findings.

Some authors have drawn parallels between ONFH in the elderly and rapidly destructive arthrosis (RDA) of the hip, a syndrome of idiopathic rapid joint deterioration seen in older women (15). In RDA, subchondral fractures and bone marrow edema are thought to drive a swift collapse of the femoral head, often within months (16). Many RDA cases historically labeled as “rapid OA” likely represent unrecognized osteonecrosis or subchondral insufficiency fractures (15). For example, in Shibayama’s series, 16.7% of elderly ONFH cases followed a course resembling rapidly destructive osteoarthritis, with the necrotic process extending to the acetabulum (4). This suggests a spectrum in the elderly from localized ONFH to widespread joint destruction, unified by an underlying failure of bone maintenance.

Demographically, elderly ONFH exhibits a different pattern compared to traditional ONFH. In younger cohorts, there is a male predominance, especially in alcohol-related cases, and bilateral hip involvement is common (more than 50% in steroid-induced ONFH) (17). In contrast, reports suggest that spontaneous ONFH or subchondral fractures in the elderly may have a female predominance, likely reflecting the higher incidence of osteoporosis in older women (18). Bilateral involvement in elderly idiopathic cases is less frequent, although when systemic factors like steroid exposure or metabolic disease are present, multiple joints can be affected (19).

The overall incidence of elderly ONFH remains unclear; one Japanese study noted that idiopathic ONFH over age 60 was rare but progressed rapidly when present (4). The aging global population and improved survival of patients with chronic diseases (20) (often corticosteroid-treated) suggest clinicians may encounter senescence-driven ONFH more frequently. Population studies link ONFH with age-related comorbidities. A Taiwan national database study found diabetes mellitus associated with 1.16-fold increased ONFH risk after adjustment (21). Though modest, this finding is notable given diabetes prevalence increases with age (13), suggesting metabolic derangements in older adults contribute to ONFH risk, consistent with vascular and cellular senescence acceleration by diabetes (22). Similarly, ONFH patients show higher cardiovascular event incidence than age-matched controls (5). A large cohort study reported significantly elevated major cardiovascular/cerebrovascular events in ONFH patients (19% vs. 14% controls) with 1.3-fold higher adjusted risk (5). This association persisted in young ONFH patients after controlling for traditional risk factors, indicating shared pathways like endothelial dysfunction between ONFH and atherosclerotic disease (5). In elderly populations, individuals with cardiovascular risk factors (hypertension, hyperlipidemia) or events may face heightened osteonecrosis risk due to compromised blood flow or pro-thrombotic tendencies.

In summary, although epidemiological data on senescence-driven ONFH are limited, evidence suggests it is underdiagnosed and mechanistically distinct. The typical elderly ONFH patient likely has occult risk factors (mild osteopenia, vascular risk, subclinical steroid exposure) and develops hip pain initially attributed to degenerative disease. Delayed diagnosis until collapse is characteristic, when joint-preserving options are limited. Recognizing this pattern is crucial for improving outcomes: if clinicians consider ONFH in differential diagnosis of atypical elderly hip pain and employ early MRI or bone scan imaging, more cases could be detected when interventions might prevent collapse. This need for heightened awareness leads to exploring how aging creates conditions favoring femoral head osteonecrosis and how these insights could inform screening and prevention. To underpin Table 1 with a reproducible evidence base, we conducted a targeted, systematic search of PubMed/MEDLINE and Web of Science (last search: 1 October 2025) to identify original clinical studies reporting the natural history or treatment outcomes of osteonecrosis of the femoral head (ONFH) or subchondral insufficiency fractures (SIF) in older adults. The search strategy combined condition- and population-specific terms as follows: (“osteonecrosis of the femoral head” OR “avascular necrosis” OR “ONFH” OR “AVN” OR “subchondral insufficiency fracture” OR “SIF”) AND (“elderly” OR “aged” OR “older adults” OR “geriatric”).

**Table 1:** Clinical outcomes and study characteristics in elderly-predominant ONFH and SIF cohorts.

Study (Year), Country	Design	Cohort (hips/patients)	Mean age (years)	Female (%)	Baseline definition	Follow-up duration	Outcome definition	Collapse/progression rate (%)	THA rate (%)	Risk of bias
Shibayama (2000), Japan (4)	Retrospective Series	18 patients (hips NR)	≥60	NR	Idiopathic ONFH (histologically confirmed)	NR (short-term)	Radiographic progression (collapse, JSN, RDA)	66.7% (Collapse) 22.2% (RDA)	NR	High
Yoon et al. (2014), Korea (137)	Retrospective Series	31 hips/31 patients	68.9	74.2%	SIF (radiographically confirmed)	NR	FHC progression >2 mm; THA for intractable pain	NR	48.4%	Unclear
Hackney et al. (2015), USA/Korea (84)	Retrospective Series	35 hips (with outcome)	NR (Mixed-age)	NR	SIF (MRI-confirmed)	~6 months (mean time to THA)	Conversion to THA	NR	45.7%	Unclear
Ikemura et al. (2016), Japan (154)	Retrospective Series	15 hips/14 patients	66	64.3%	SIF with BME location	NR	Progression to RDA	20.0% (RDA)	NR	High
Shimizu et al. (2020), Japan (155)	Retrospective Series	44 hips/41 patients	74.1	82.9%	SIF (MRI-confirmed)	NR	Conversion to THA	NR (JSW narrowing reported)	34.1%	Unclear

BME, bone marrow edema; FHC, femoral head collapse; JSN, joint space narrowing; JSW, joint space width; NR, not reported; ONFH, osteonecrosis of the femoral head; RDA, rapidly destructive arthrosis; SIF, subchondral insufficiency fracture; THA, total hip arthroplasty. Study selection and data items follow the embedded methods described in the Epidemiology and Diagnostic Challenges section. Only original clinical studies are included. When elderly-only data were not isolatable [e.g., Hackney et al. (84)], the mixed-age cohort was retained with this limitation noted, as age was a significant predictor of THA in their analysis. Risk of bias reflects a simplified screen based on key Methodological Index for Non-Randomized Studies (MINORS) criteria (e.g., clear aim, retrospective data collection, unbiased assessment, appropriate follow-up). Given the prevalence of small, retrospective, single-center designs for this specific clinical question, the overall evidence quality is limited, and risk is commonly rated as “Unclear” or “High”. “Collapse/Progression Rate” is a composite endpoint representing the most severe outcome reported, including FHC, progression to RDA, or significant JSW narrowing, as defined by the individual studies.

Inclusion criteria were: (1) original clinical research (cohort studies or case series with *n* ≥ 10 hips); (2) mean or minimum cohort age ≥60 years, or an analyzable elderly subgroup; (3) quantitative reporting of femoral head collapse and/or conversion to THA; and (4) English language. Exclusion criteria were: reviews, editorials, letters, conference abstracts, animal/biomechanical studies, and series with <10 hips. For each included study, we extracted sample size, mean age, baseline disease definition/stage (as reported), follow-up duration, definitions of collapse/outcome, and rates of collapse and THA. Study quality was screened using key MINORS items (clear aim, consecutive inclusion, prospective vs. retrospective data, unbiased assessment, appropriate follow-up), summarized as a simple low/unclear/high risk-of-bias indicator.

**Figure 1:**
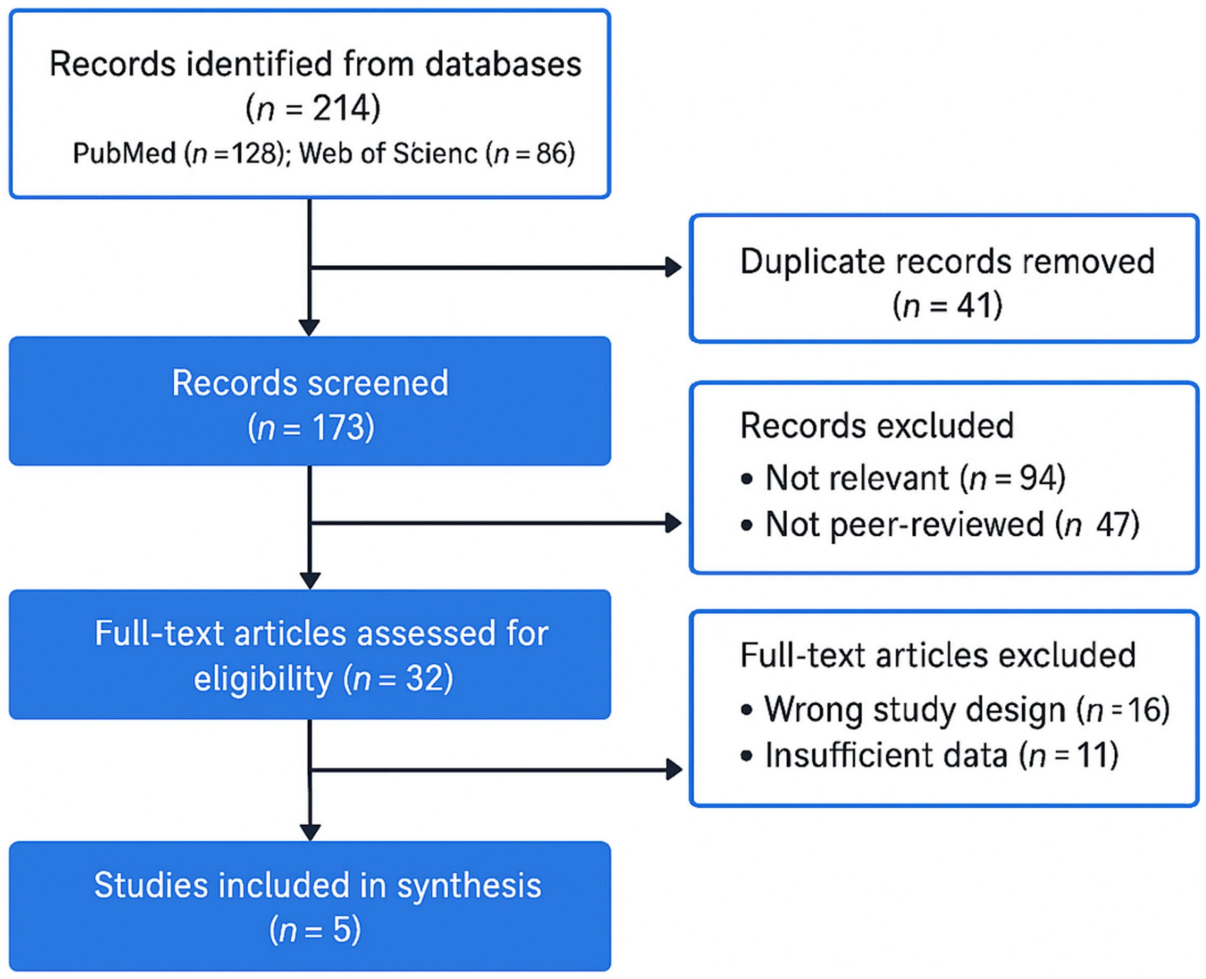
PRISMA flow diagram of the literature search and study selection process.

The study selection process is summarized in the PRISMA flow diagram (Figure 1). PubMed and Web of Science (to 1 Oct 2025) yielded 214 records (PubMed 128; WoS 86); 41 duplicates were removed, leaving 173 titles/abstracts screened and 141 excluded (reviews/editorials/abstract-only 67; non-hip/pediatric 14; animal/biomech/nonclinical 24; insufficient elderly focus 36). Thirty-two full texts were assessed; 27 were excluded (*n* < 10 hips 6; no quantitative collapse/THA 11; elderly subgroup not isolatable 8; cohort overlap 2). Five original studies were included (Table 1). Despite small samples and heterogeneous definitions, the synthesized evidence consistently indicates that elderly-predominant SIF/ONFH exhibits a rapidly progressive, structurally vulnerable phenotype distinct from typical younger ONFH.

## Role of systemic aging processes: vascular senescence marrow adiposity and “inflammaging”

Aging involves cumulative physiological changes affecting all organ systems, with bone and vascular alterations particularly predisposing to osteonecrosis. Key interrelated factors include endothelial senescence and vascular aging, bone marrow adiposity and reduced perfusion, and inflammaging with impaired osteogenic repair. These aging-related changes create a vulnerable femoral head environment prone to ischemic injury and structural failure.

### Endothelial senescence and vascular fragility

Age-related vascular changes compromise femoral head blood supply. The femoral head receives blood primarily from lateral epiphyseal branches of the medial circumflex femoral artery through the femoral neck (23). While younger individuals maintain adequate perfusion with compensatory capacity (24), aging causes documented declines in endothelial function and microvascular density (25). Endothelial cells undergo senescence—permanent cell cycle arrest with pro-inflammatory secretions—following oxidative stress and DNA damage, manifesting as reduced angiogenic capacity and diminished vasodilatory response (26). Concurrent atherosclerosis narrows upstream arteries, further increasing ischemia risk.

Age-related marrow adiposity reduces femoral head perfusion. Bluemke et al.’s MRI perfusion study demonstrated that femoral head perfusion (gadolinium enhancement rate) inversely correlated with marrow fat content (6). Older subjects with higher marrow fat showed correspondingly lower perfusion, suggesting age-related marrow adiposity diminishes subchondral bone microcirculation (27). Patients on long-term steroids without ONFH had higher perfusion than controls, hinting that robust microcirculation may protect against osteonecrosis despite systemic risk factors (6). In elderly individuals, baseline low perfusion means normal activities may induce relative femoral head hypoxia.

Aging-related vascular dysfunction contributes to ONFH through systemic and local mechanisms. Clinical studies show ONFH patients have significant associations with major cardiovascular events, suggesting endothelial dysfunction as a common pathway (5). In aging, endothelial dysfunction includes reduced nitric oxide bioavailability and heightened endothelin-1 activity, promoting vasoconstriction and thrombosis in intraosseous vessels (28). Such changes in the intraosseous vessels could predispose the bone to thrombotic occlusion or inability to auto-regulate under stress. Furthermore, microvascular networks undergo age-related rarefaction (7), with histomorphometric studies showing decreased capillary density in older bone marrow (8). Although data specific to the femoral head are scarce, it is reasonable to extrapolate that an elderly femoral head has fewer functional capillaries and a lower reserve to adapt to occlusion.

Aging impairs post-ischemic angiogenic repair mechanisms. Young animals and patients can sometimes heal small osteonecrotic lesions by creeping substitution: new blood vessels invade the necrotic zone and bring in reparative cells (29). In aging, endothelial cells exhibit reduced proliferative capacity and decreased growth factor secretion (30). Key regulators like VEGF are downregulated in aged tissues due to cellular senescence and reduced hypoxia signaling (31). Notably, type H endothelium—specialized bone capillaries coupling angiogenesis with osteogenesis—decline markedly with aged (32). Kusumbe et al. demonstrated that these regenerative endothelial cells supporting perivascular osteoprogenitors are significantly reduced in aged mouse bones; however, pharmacological angiogenesis stimulation partially restored bone mass (33). In humans, aged femoral heads may experience both increased ischemia susceptibility and impaired post-infarct revascularization, creating a “double hit” scenario favoring progression to collapse.

### Marrow adiposity and mechanoadaptation failure

Skeletal aging involves a compositional shift from hematopoietic (red) to fatty (yellow) marrow (34). By age 70, the proximal femur contains abundant adipocytes within marrow spaces, creating significant biomechanical and metabolic consequences (35). While adipocytes secrete bioactive adipokines, excessive marrow fat reduces bone formation and displaces hematopoietic and stromal elements essential for osteoblast and vascular support (36). In ONFH, marrow adiposity increases both ischemic susceptibility and structural weakness.

Increased marrow fat correlates with reduced perfusion. Fat cells require less oxygen than hematopoietic tissue, and fat-laden marrow demonstrates decreased blood flow as vascular networks regress when less needed for hematopoiesis (37–39). Additionally, intramedullary fat expansion elevates intramedullary pressure. In steroid-induced osteonecrosis, fat hypertrophy raises bone marrow pressure, compressing vessels and causing ischemia (40). Similarly, elderly patients experience gradually rising marrow pressure and reduced capillary density, creating chronic borderline perfusion in the femoral head. Under this condition, even a mild reduction in systemic blood pressure or a small embolic event could precipitate localized necrosis (41).

Marrow adiposity reflects altered lineage allocation in bone marrow mesenchymal stem cells (BMSCs). With aging, BMSCs preferentially differentiate into adipocytes rather than osteoblasts, impairing osteogenesis and trabecular bone maintenance (42). In younger bone, mechanical loading signals BMSCs toward osteoblastic differentiation (43). In older bone, fewer progenitors exist and are biased toward adipogenic differentiation, resulting in impaired mechanoadaptation where normal remodeling responses are blunted, allowing microcracks to accumulate (42, 44). Over time, the elderly femoral head subchondral plate becomes riddled with microscopic damage and diminished thickness (45). Finite element analyses and micro-CT studies show decreased trabecular connectivity and heterogeneous mineralization, indicating stress concentration areas (46).

These changes predispose to subchondral insufficiency fractures (SIF), occurring under normal physiological stress in mechanically compromised bone. SIF frequently affects osteoporotic elderly women, presenting as acute hip pain and mimicking ONFH on imaging (47, 48). In fact, SIF and ONFH are difficult to distinguish: SIF appears as irregular subchondral low-intensity bands on MRI, while classical ONFH shows smoother, concave demarcation lines (49). However, these entities often overlap: insufficiency fractures can disrupt vasculature causing secondary osteonecrosis, while osteonecrotic areas weaken bone predisposing to fracture (48). In elderly patients, it may not matter which came first; the end result is a necrotic, collapsed femoral head. The key point is that the aged subchondral bone is mechanically fragile. Even without a discrete fracture line, microdamage can accumulate to a point where the bone cannot support the overlying articular cartilage, leading to a collapse often seen as the crescent sign in ONFH (50). Histologic analyses reveal severe cartilage degeneration and collapsed necrotic trabeculae extending damage to adjacent viable bone (4). The absence of sclerotic borders suggests overwhelmed reparative responses due to reduced osteoblast activity and ongoing mechanical stress (51).

In summary, marrow adiposity and declining mechanical competence are central aging processes facilitating osteonecrosis. Aged bone is inadequately constructed and repaired, failing under stresses tolerated by younger bone. Such failure further compromises blood flow through fracture or edema-induced intraosseous pressure elevation, creating a vicious cycle. This contrasts with younger ONFH patients who typically have normal bone quality unless compromised by specific treatments (e.g., prolonged steroids causing osteoporosis). In elderly patients, poor bone quality effectively lowers the threshold for ONFH occurrence.

### Inflammaging and impaired osteogenesis–angiogenesis coupling

Aging involves chronic low-grade inflammation termed “inflammaging,” characterized by elevated pro-inflammatory cytokines (IL-6, TNF-*α*, CRP) even without overt disease (52). This systemic inflammatory milieu detrimentally affects bone maintenance by promoting osteoclast activity while inhibiting osteoblast differentiation, tilting the balance toward bone resorption in elderly femoral heads and exacerbating osteonecrotic progression (53).

Moreover, chronic inflammation can impair the coupling of angiogenesis to osteogenesis. During bone regeneration (such as after a microfracture or an infarct), there is a coordinated sequence where new blood vessels and new bone form together (54). Inflammation is a double-edged sword in this context: acute inflammation is necessary to initiate healing (55), but chronic inflammation can derail the process (56). A persistent inflammatory environment may lead to fibrosis instead of proper bone repair (57), or to premature apoptosis of the progenitor cells needed for regeneration (55). There is evidence that aging shifts macrophage polarization toward pro-inflammatory (M1) rather than healing (M2) responses during bone repair, resulting in prolonged inflammatory mediator release that inhibits angiogenesis through mechanisms like TNF-*α*-induced endothelial cell apoptosis (58).

In ONFH, the transitional zone between necrotic and viable bone represents the primary repair site (12). Recent studies demonstrate heavy infiltration of senescent cells secreting inflammatory cytokines in this zone, particularly in ischemic necrosis cases (59). Okamoto identified strong senescence-associated *β*-galactosidase activity and elevated p16INK4a/p21 expression in border cells of necrotic human femoral heads (59). These senescent cells, localized in trabecular bone and marrow, exhibit a senescence-associated secretory phenotype (SASP) with elevated IL-6 and degradative enzymes (e.g., MMP-3) (59). The presence of senescent osteoblasts/osteocytes and mesenchymal stromal cells suggests impaired bone repair, as these cells secrete inflammatory/degradative factors rather than promoting regeneration (60). Inflammaging likely predisposes elderly bone marrow to such outcomes, as pre-existing senescent cells from lifelong replicative stress undergo additional senescence waves when osteonecrosis triggers cellular stress (61).

Angiogenesis-osteogenesis coupling represents another critical aspect of bone repair. Normal bone formation requires tight coordination with new vessel formation through molecular pathways including VEGF and HIF-1α (62). Aging compromises both components: reduced osteoprogenitors (63) and diminished angiogenic signals (64). Specialized type H vessels in bone produce factors stimulating osteoprogenitors (64); their age-related loss results in fewer maintained osteoprogenitors (63). Additionally, new vessels formed in aged tissues are often abnormal (leaky or sparse) (65), rendering repair attempts in elderly ONFH ineffective due to uncoupled or insufficient angiogenesis-osteogenesis. This dysfunction explains the absent sclerotic margin in many elderly ONFH cases. In younger patients, sclerotic rims result from viable bone attempting to wall off necrotic areas through coupled angiogenesis/osteogenesis, whereas this process fails in older patients, causing diffuse bone loss without clear demarcation (4).

In summary, aging’s systemic pro-inflammatory state and declining regenerative signaling diminish ONFH healing capacity. Once necrosis initiates in aged bone, it propagates rather than being contained. Senescent cells at lesion sites further exacerbate inflammation and tissue degradation. The concept of “sterile inflammation” driving osteonecrotic bone loss aligns with these observations—without infection or autoimmune disease, aged cells create inflammatory microenvironments that perpetuate tissue destruction.

**Figure 2:**
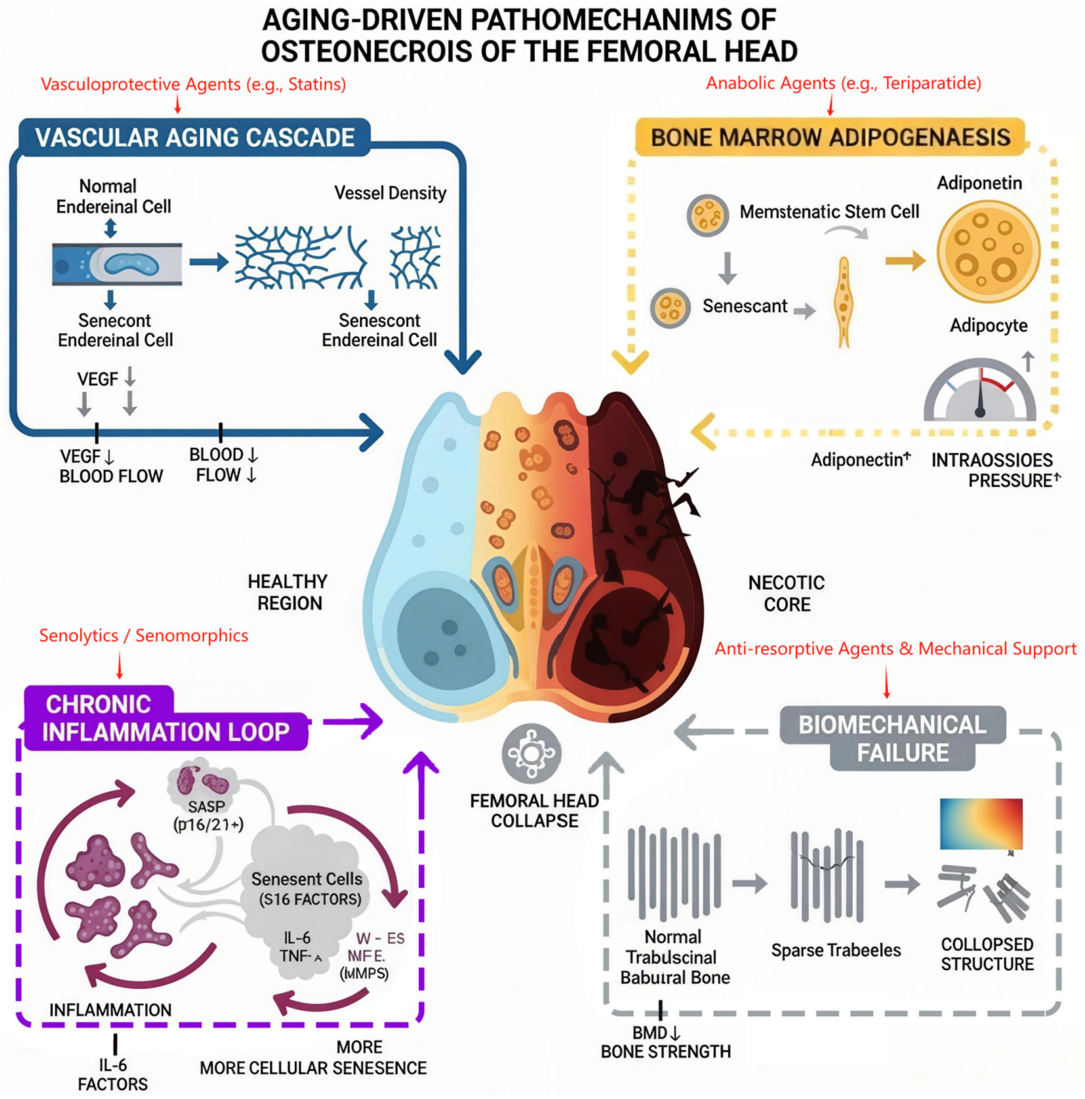
A conceptual model of senescence-driven pathogenesis in ONFH.

These interconnected aging processes—vascular senescence, marrow adiposity, chronic inflammation, and biomechanical failure—converge to create the pathogenic conditions that predispose the elderly femoral head to osteonecrosis. Figure 2 provides a schematic overview of this proposed model, illustrating how these four key pathological cascades culminate in femoral head collapse, transitioning from a healthy state to a necrotic core.

The model integrates four key age-related pathological cascades that converge to drive ONFH progression. Among these, the “Chronic Inflammation Loop” is substantiated by direct molecular evidence from human ONFH tissue; in this pathway, senescent cells perpetuate a pro-inflammatory state via their secretory phenotype (SASP), impairing tissue repair. In parallel, the model posits three interconnected pathways extrapolated from fundamental aging biology. Vascular aging is proposed to reduce critical blood flow, while bone marrow adipogenesis is thought to increase intraosseous pressure, further compromising perfusion. Ultimately, these processes contribute to biomechanical failure, where the deteriorating bone microarchitecture can no longer withstand physiological loads. The convergence of these evidence-based and hypothesized pathways provides a comprehensive framework for the progression from a healthy femoral head to a necrotic and collapsed state.

## Common age-related comorbidities as catalysts

Aging rarely occurs in isolation from other health conditions. Comorbidities prevalent in elderly populations can amplify pathological processes leading to ONFH, with diabetes mellitus, osteoporosis, and cardiovascular disease deserving particular attention.

### Diabetes mellitus

Diabetes causes systemic microangiopathy, further impairing the already compromised femoral head blood supply. Hyperglycemia drives advanced glycation end-product (AGE) formation, stiffening bone collagen and reducing bone quality. Clinically, diabetes represents an independent ONFH risk factor (21). While risk increases are moderate, diabetes may convert subclinical osteonecrosis into progressive disease by inhibiting compensatory mechanisms. Diabetic patients exhibit higher marrow fat content (66) and exaggerated SASP due to metabolic stress (67)—effectively “accelerated aging”—resulting in healing-refractory ONFH. Additionally, diabetic dyslipidemia promotes fat emboli and thrombi formation (68), potentially occluding femoral head vessels analogous to steroid-induced fat embolism.

### Osteoporosis

Many elderly individuals, particularly postmenopausal women, develop osteopenia or osteoporosis (69). Low bone mineral density and deteriorated microarchitecture directly increase insufficiency fracture risk. In ONFH contexts, osteoporosis reduces femoral head weight-bearing capacity. SIFFH strongly associates with osteoporotic bone (9, 70–72). Even without complete fractures, osteoporotic bone has fewer trabeculae for load distribution, creating higher stress on remaining bone and potentially enlarging necrotic areas through mechanical failure of adjacent trabeculae (73). While debate exists regarding whether osteoporosis per se predisposes to ONFH, it likely contributes indirectly through this mechanism. Osteoporosis treatments may influence ONFH outcomes: bisphosphonates reduce bone turnover and may preserve subchondral bone in early ONFH, with studies showing delayed collapse (74). However, severely suppressed turnover could theoretically impair creeping substitution of necrotic bone, highlighting that altered bone density and remodeling rates in elderly patients are integral to the ONFH equation (75).

### Cardiovascular disease

Elderly individuals frequently have coronary artery disease, peripheral vascular disease, or stroke histories, reflecting systemic atherosclerosis and hypercoagulable states that potentiate osteonecrosis. Medial femoral circumflex artery atherosclerosis could reduce baseline femoral head flow (76), analogous to carotid stenosis reducing cerebral perfusion reserve. Atrial fibrillation or clotting tendencies may cause small emboli to occlude femoral head branch arteries. Age-related coagulability increases compound this risk, as elderly vessels are narrower and less tolerant of blockages (77). ONFH association with major cardiovascular events suggests macro- and micro-circulatory issues are interconnected (5). Lipid metabolism represents another cardiovascular factor: statin use links to decreased steroid-related ONFH incidence (78), presumably by reducing bone lipid deposition and improving endothelial function, while severe hyperlipidemia may increase risk through fat embolization or increased marrow fat content.

Essentially, comorbidities serve as catalysts exacerbating intrinsic aging processes by adding vascular insufficiency (diabetes, atherosclerosis), mechanical weakness (osteoporosis), or both. This synergy may explain why some elderly patients without classic risk factors develop ONFH—aging provides fertile ground, and common comorbidities become the proverbial “straw that breaks the camel’s back.

## Imaging phenotypes in the elderly: distinct patterns and evolution

Imaging provides insight into ONFH pathological processes and reveals differences between senescence-driven lesions and those in younger patients. In elderly patients, radiographic and MRI features often reflect underlying ischemia-insufficiency fracture combinations and rapid progression.

### X-ray

**Figure 3:**
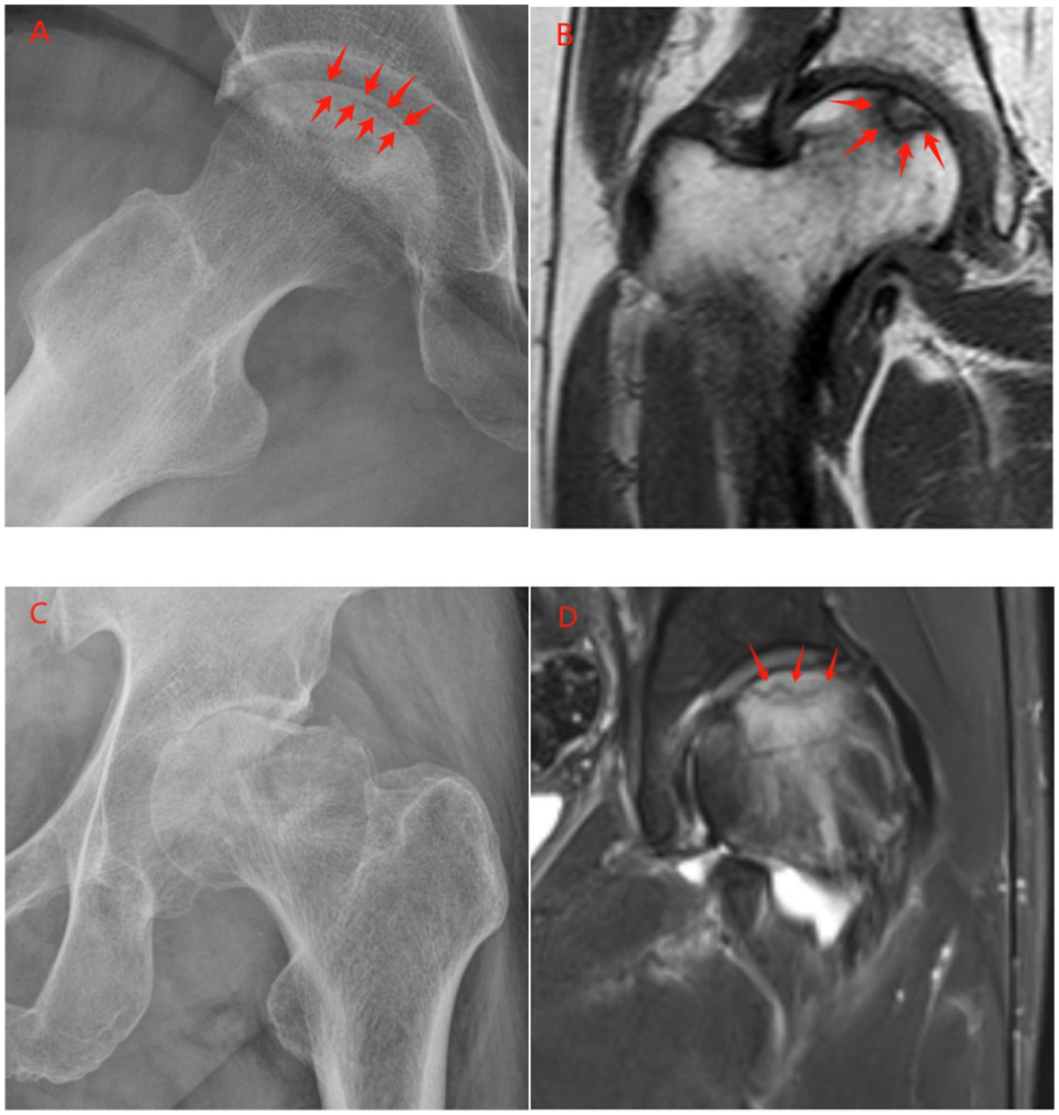
Radiographic and MRI comparison of classic versus senescence-driven ONFH. **(A)** Anteroposterior frog-leg radiograph of a 66-year-old female with classic ONFH, showing a subchondral crescent sign (arrow) indicating early collapse, surrounded by a distinct sclerotic border. **(B)** Coronal T1-weighted MRI from the same patient demonstrates a well-defined, low-signal intensity band (arrow) demarcating the necrotic lesion. **(C)** In contrast, this anteroposterior radiograph of a 66-year-old male with senescence-driven ONFH shows rapid destruction and resorption of the femoral head with severe joint space narrowing, characteristic of a rapidly destructive course. Note the absence of a clear sclerotic margin. **(D)** Coronal fat-suppressed T2-weighted MRI of a 61-year-old male, illustrating features within the senescence-driven ONFH spectrum. It reveals an irregular low-intensity fracture line (arrow) and extensive, high-signal bone marrow edema (arrowheads) throughout the femoral head, a stark contrast to the well-demarcated lesion in classic ONFH.

Early ONFH (pre-collapse) may show unremarkable X-rays regardless of age (79). However, post-subchondral collapse patterns can suggest elderly-type ONFH. While younger ONFH typically displays the crescent sign—a subchondral linear lucency representing fracture planes between necrotic and intact bone with sclerotic margin (Figure 3A) (2)—elderly patients may show irregular or ill-defined crescent signs (80). Rather than discrete dead bone sequestra, X-rays may demonstrate diffuse demineralization and rapid joint space loss resembling severe osteoarthritis (81). Shibayama’s study noted progressive collapse within short periods in two-thirds of elderly ONFH cases, with one-third showing rapid joint space narrowing following collapse (4). Approximately 22% exhibited “rapid destruction and resorption” with femoral head fragmentation and disappearance over months, resembling rapidly destructive arthrosis (Figure 3C) (4). Notably, 16.7% had acetabular extension, though milder than typical RDA (4). The absence of robust demarcation sclerosis in most elderly cases (4) represents an imaging correlate of poor reparative response, contrasting with younger patients who often show reactive sclerotic rings attempting lesion containment.

### MRI

MRI remains the gold standard for early ONFH detection, providing detailed marrow change visualization (82). In younger at-risk individuals, MRI detects stage I ONFH (pre-radiographic) via characteristic “double-line signs” on T2-weighted images and T1 low-signal bands corresponding to necrotic-reactive bone interfaces (Figure 3B) (83), while in elderly patients, MRI sensitivity remains high, but osteoarthritic changes and osteoporotic marrow complicate interpretation. Key MRI findings in SIF include irregular, non-convex T1 low-intensity bands with adjacent bone marrow edema (Figure 3D) (84, 85), contrasting with classic ONFH’s smooth, concave bands following necrotic segment shapes (49). Notably, many elderly patients exhibit both patterns: irregular fracture lines and necrotic segments (86). Bone marrow edema (BME) patterns are particularly diagnostic, as in ONFH, extensive femoral head and neck BME indicates impending or evolving collapse (87), whereas in SIF, BME is nearly universal as fracture reaction (88). Kenyu et al. found BME absence in femoral head SIF associated with collapse progression, suggesting inadequate biological responses predict poor outcomes—equally applicable to elderly ONFH where edema absence might indicate “cold” bone death with poor healing (89). Furthermore, MRI tracks lesion evolution effectively, as younger patients’ early-detected ONFH may remain stable or partially revascularize, while elderly follow-up MRIs often show rapid necrotic volume increases and articular surface involvement (90). Serial imaging may reveal subchondral crack conversion to complete femoral head dome collapse (91). Additionally, acetabular status on MRI is notable: rapidly destructive cases show acetabular edema and cystic changes (92), whereas typical ONFH remains femoral head-confined until very late stages, with early acetabular reaction suggesting particularly aggressive processes common in elderly patients (93).

### CT

CT scans, less common for early diagnosis, help visualize fracture lines and collapse extent (94). In elderly patients with hip pain, CT may rule out fractures when MRI is contraindicated. CT demonstrates subchondral fractures as linear defects and detects small collapse areas. For ONFH vs. SIF differentiation, Uchida et al. used CT to identify fracture lines in suspected SIF (95). Elderly ONFH CT may show more comminuted collapse (multiple subchondral bone fragments) versus single sequestra in younger patients (96). Sclerosis patterns also differ: younger patients show clear demarcation sclerosis lines, while older patients may display patchy or absent sclerosis (96).

### Bone scintigraphy and PET

When MRI is unavailable, Technetium-99 m bone scans or PET detect early ONFH, typically showing central cold spots (photopenia) where blood flow is absent, surrounded by increased uptake rings (reactive interfaces) (97). In older patients, bone scans may be less useful due to concomitant arthritic changes causing tracer uptake, complicating interpretation (98). However, PET scans examining perfusion and cellular activity may help in ambiguous cases. PET or SPECT can detect early AVN stages and predict prognosis (95). Poorly perfused femoral heads on SPECT in older patients with hip pain should raise imminent collapse concerns, reinforcing that low perfusion areas correlate with ONFH severity (41).

### Imaging summary

Senescence-driven ONFH imaging phenotypes often combine osteonecrosis with insufficiency fracture features. Rapid sequential image progression represents a red flag, as does lack of clear reparative response (minimal sclerosis, extensive bone loss) (4). Shibayama’s research effectively demonstrated elderly patients’ hip X-rays progressing from mild flattening to near-complete femoral head destruction with superior migration rapidly (4). Such dramatic changes are uncommon in younger patients unless particularly malignant disease courses or concurrent infections occur. Therefore, radiologists and clinicians should recognize that elderly patient “ONFH” is not benign—it tends to predict rapid collapse. Early MRI is warranted for equivocal X-rays. Distinguishing primary ONFH from subchondral fracture on MRI may be academic because management overlaps, but if a fracture line is seen (irregular band), one should offload the joint immediately as one would for a fracture, in hopes of preventing displacement.

What we describe as “elderly ONFH” may often represent subchondral insufficiency fracture radiologic entities. SIFFH literature emphasizes prevalence in osteoporotic elderly and differentiation needs from ONFH (47, 99). However, therapeutically and conceptually, both lead to necrosis and collapse. In elderly patients, thinking in continuum terms may be most accurate: osteoporotic bone yields (fractures) with supervening ischemia, or ischemia occurs with bone yielding—either way resulting in combined pathology. Imaging confirms ongoing processes and gauges extent.

## Molecular and cellular insights: senescence markers, gene expression, and cell death in elderly ONFH

Advancements in molecular biology and “omics” technologies have illuminated pathological mechanisms underlying ONFH, particularly aging influences. Key findings regarding cellular senescence markers, angiogenesis/apoptosis-related gene expression, and omics data support the concept of senescence-driven ONFH.

### Cellular senescence in ONFH: direct evidence

The most compelling evidence linking aging biology to ONFH comes from demonstrating senescent cells in osteonecrotic femoral heads. Okamoto et al. examined femoral head samples from ONFH patients, discovering senescent cell accumulation at necrotic lesion borders (59). These cells expressed classical senescence markers p16INK4a and p21, with DNA damage foci (*γ*-H2AX positivity) indicating genomic stress (59). Significantly, senescent cells encompassed multiple cell types: mesenchymal stem/stromal cells (nestin-positive), osteoblastic cells (periostin-positive), and osteocytes (DMP-1-positive), suggesting entire bone cellular network senescence in affected regions (59). This implies premature local bone tissue aging responding to necrotic injury or contributing to injury causation. While healthy aged bone shows increased baseline senescent osteocytes and stromal cells compromising bone quality, ONFH findings might represent exacerbation where ischemic stress accelerates cellular senescence.

The secretory profile of senescent cells proves equally significant. Elevated IL-6 in transitional zone marrow aligns with senescence-associated secretory phenotype (SASP) (100). Upregulated RANKL could promote osteoclast activity and bone resorption around necrotic areas (101). Elevated MMP-3 (matrix metalloproteinase-3) can degrade extracellular matrix and cartilage, contributing to joint destruction (102). These molecular signals link cell senescence to aggressive ONFH progression in elderly patients: senescent cells actively create microenvironments that break down tissue and impair regeneration rather than remaining inert bystanders.

Therapeutically, Okamoto et al. demonstrated that human mesenchymal stem cell-conditioned medium (MSC-CM) in rodent ONFH models reduced senescent cell burden in necrotic bone and significantly preserved trabecular bone volume, preventing collapse (59). While not strictly senolytic, the MSC secretome likely modulated SASP or encouraged cellular rejuvenation (103). This suggests tantalizing possibilities for targeting senescence in ONFH treatment—an approach particularly beneficial for older patients with high senescent cell burdens.

To further substantiate these histological observations with functional mechanisms, recent studies have focused on patient-derived cells. Tang et al. (104) demonstrated that BMSCs isolated directly from ONFH patients exhibit a premature senescence phenotype driven by the miR-601/SIRT1 axis. This functional impairment in human cells provides a critical mechanistic bridge, confirming that the senescence pathways identified in animal models are actively recapitulated in human pathology.

### Aging-related gene expression in ONFH

Gene expression studies reveal aging’s role in ONFH pathogenesis, as demonstrated by Qiu who specifically examined aging-ONFH intersections in steroid-induced cases (105). Using peripheral blood gene expression from steroid-induced ONFH patients cross-referenced with known aging-related genes, they identified 41 dysregulated aging-related genes enriched in oxidative stress response, FoxO signaling, and TNF signaling pathways (105). Among the key highlighted genes were CAT (Catalase) and FOXO3, which are classic longevity genes involved in oxidative damage mitigation and stress resistance, along with IRS2 (Insulin receptor substrate 2) involved in insulin signaling and energy metabolism, and MAP2K11, part of MAPK pathway transmitting stress signals linked to inflammatory responses (105). Notably, FOXO3, a transcription factor helping cells survive oxidative stress, when altered in ONFH patients, suggests femoral head cells are less equipped to handle oxidative insults, becoming more prone to apoptosis or senescence (106). Furthermore, IRS2 involvement might relate to metabolic aspects, possibly linking diabetes or marrow adipogenesis (107). The fact that these aging-related molecules were flagged in steroid ONFH supports the idea that even steroid-induced ONFH (traditionally seen in younger populations) involves aging biology mechanisms, while for elderly ONFH, these aging pathways are likely even more pronounced.

### Cell death modalities: apoptosis vs. necrosis

Osteocyte apoptosis patterns vary by ONFH etiology. A 2010 study found steroid- and alcohol-induced ONFH had significantly higher TUNEL-positive (apoptotic) osteocytes compared to post-traumatic or idiopathic ONFH (17). This implies that systemic factor etiologies (steroids, alcohol) involve programmed cell death pathways (apoptosis) versus purely necrotic death. Idiopathic ONFH had lower apoptosis in that study (17), perhaps indicating more direct necrosis or a slower process allowing for some remodeling. If many idiopathic ONFH in their cohort were older patients, one interpretation could be that in older patients the bone dies more “quietly” (through necrosis or insufficient repair) rather than the dramatic apoptosis triggered by toxic or metabolic insults in younger patients (17). However, once collapse happens, both will end up with empty lacunae (dead osteocytes) (108). The apoptotic versus necrotic modality influences initial inflammatory responses—apoptosis generates distinct immunological signals compared to necrosis (109). Lower apoptotic indices in idiopathic cases may correlate with absent demarcation lines, reflecting diminished inflammatory and healing responses (110). Thus, cell death mechanisms appear age/risk-factor dependent: younger, risk-factor-driven ONFH involves osteocyte apoptosis (potentially targetable by cell survival interventions) (111), while older spontaneous ONFH reflects gradual ischemic cell death (112).

Studies examining VEGF, PDGF, and other gene expression in ONFH patients generally find reduced growth factor expression in necrotic areas (113), while surrounding areas show increased expression reactively (114). Aged patients have lower baseline growth factor levels. HIF-1α (hypoxia-inducible factor), crucial for upregulating VEGF in hypoxic conditions, shows dampened responses in older adults (115). Animal models demonstrate that overexpressing HIF-1α in bone cells can rescue blood flow and bone formation in steroid-induced ONFH (116–118). Translating this, elderly patients might benefit less from natural HIF-VEGF responses when ONFH begins, requiring therapeutic assistance to mount angiogenic responses.

In summary, the molecular signatures emerging from research align with the narrative that ONFH in the elderly is biologically “geriatric” at the cellular level. Senescent cells, chronic oxidative stress, impaired angiogenesis, and altered cell death patterns all point toward an environment like that of an aged tissue failing to regenerate. These insights not only validate the concept of senescence-driven ONFH but also point to specific molecular targets (senescence pathways, oxidative stress, etc.) for potential intervention.

## Biomechanical deterioration of the aging femoral head

The femoral head’s structural integrity determines whether bone necrosis progresses to catastrophic collapse or remains stable. Aging weakens the femoral head’s subchondral bone and supporting trabeculae through multiple converging changes, including reduced osteoblast function and microcrack accumulation, creating unique biomechanical risk factors in the elderly.

### Subchondral cortex and trabecular bone thinning

Age-related thinning and increased porosity of the subchondral cortical plate elevates stress per unit area on underlying bone, as this load-distributing structure becomes compromised (119). Trabecular bone architecture deteriorates through loss of horizontal cross-linking struts, leaving predominantly vertical trabeculae with increased spacing (120, 121). Research on trabecular microarchitecture demonstrates that the vertical tissue fraction explains approximately 83% (*r*^2^ = 0.83) of the variation in bone strength (120). Consequently, the femoral head transitions from a resilient shock absorber to a brittle structure. When necrosis occurs, elderly bone lacks sufficient reserve strength to redistribute loads, leading to rapid collapse around necrotic zones (122). Conversely, younger, denser bone with greater structural redundancy can tolerate small necrotic areas longer (123).

### Microdamage and fatigue failure

Decades of fatigue loading generate microdamage that normally stimulates targeted remodeling in younger individuals, where osteoclasts remove damaged areas and osteoblasts deposit new bone (124). However, aging disrupts this coordinated response; basic multicellular units (BMUs) inadequately detect and repair microcracks, allowing their coalescence under repetitive stress (125). The femoral head, subjected to millions of gait cycles, accumulates microcracks that culminate in fatigue fractures—insufficiency fractures resulting from cumulative microscopic failures rather than single traumatic events (126). Studies of femoral heads from elderly hip replacement patients reveal remarkably high microcrack densities occurring alongside a ~ 30% reduction in trabecular bone volume (BV/TV) in necrotic zones, indicating that even without osteonecrosis, elderly subchondral bone exists in precarious biomechanical balance (127).

Furthermore, elderly bone exhibits reduced fracture toughness due to altered collagen cross-linking. This is partly driven by AGE accumulation, which is associated with a significant reduction in post-yield energy to fracture (128). Consequently, aged bone is not only weaker but also more crack-prone.

### Subchondral insufficiency fracture mechanism

SIF in elderly hips provides mechanistic insights. Typically, elderly patients (often osteopenic women) develop acute hip pain following minimal activity. MRI reveals subchondral fracture lines and edema resulting from insufficiency fractures when bone cannot withstand applied loads (129). Small fractures with intact blood supply may heal if offloaded; however, femoral head fractures often cause overlying cartilage and bone segments to lose support, collapsing into the head and disrupting vasculature—essentially creating necrotic segments resembling ONFH (83). Systematic reviews of SIFFH show that despite initial non-surgical management with protected weight-bearing, approximately 35% eventually require total hip arthroplasty due to collapse and persistent pain (48). Successful outcomes correlate with smaller fractures and minimal collapse. Studies emphasize differentiating SIF from ONFH on imaging, as management differs (immediate offloading for SIF) (48). Many “senile ONFH” cases may represent SIF progressing to osteonecrosis due to impaired bone healing capacity. Biomechanically, this represents bone toughness failure. Elderly bone exhibits reduced fracture toughness due to altered collagen cross-linking (partly from AGE accumulation through glycation) and modified mineral properties (128). Aged bone is not only weaker but also more crack-prone with enhanced crack propagation.

### Cartilage and subchondral unit

Elderly joint cartilage is also aged and stress-intolerant (130). While cartilage’s role in ONFH is often overlooked until collapse-induced buckling occurs, rapidly destructive cases show parallel cartilage deterioration with bone loss (131). Shibayama observed “severe cartilage degeneration” in elderly ONFH patients (4). Poor bone-derived nutrition (subchondral bone supplies deep cartilage nutrients) or inflammatory mediators from senescent cells (like MMPs) may accelerate cartilage breakdown (132). Cartilage damage concentrates loads on bone edges, worsening fragmentation (133). Thus, the entire subchondral unit (bone + cartilage) becomes compromised in aged femoral heads, whereas younger ONFH patients maintain relatively healthy cartilage until collapse, sometimes bridging collapsed segments temporarily (12, 131).

### Comparison of mechanical vs. ischemic contributions

Comparing elderly and young ONFH cases reveals distinct pathophysiological sequences. In young steroid-induced ONFH (134): steroid administration → lipid deposition/microemboli → vessel occlusion → bone cell death (framework initially intact) → attempted repair (sclerosis rim) → eventual collapse if inadequate. In elderly cases (135): chronic bone microdamage + marginal perfusion → minor event triggers subchondral crack and vessel injury → mixed necrosis and fracture → weak repair response allows crack widening and necrosis spread → collapse. Mechanical deterioration dominates the elderly pathway (135), while vascular insult primarily drives the young pathway (134). Of course, there’s no absolute dichotomy, but emphasis differs. This distinction has therapeutic implications: mechanical support (bone grafts, prophylactic fixation) may benefit elderly patients more, whereas revascularization strategies might be more crucial in young patients, though ideally both factors should be addressed in all cases.

In conclusion, the aging femoral head can be thought of as a structure with reduced load-bearing capacity and reduced self-repair capacity. When osteonecrosis occurs, it behaves like an old dam developing cracks—likely to fail catastrophically due to generalized material degradation. These biomechanical insights emphasize early intervention importance: detecting lesions before gross collapse may enable bone bolstering options (bone cement injection, bone substitutes, internal fixation devices) to prevent collapse.

### Comparison with non-elderly ONFH: shared and distinct features

To further clarify why we consider senescence-driven ONFH a distinct entity, it is helpful to juxtapose it with ONFH in younger patients (those in their 20s–50s) across various dimensions: etiology, pathology, clinical progression, and treatment response. Table 2 provides an overview of the contrasting features between typical non-elderly ONFH and elderly ONFH (senescence-driven), as distilled from the preceding discussions.

**Table 2:** Comparison of typical osteonecrosis of the femoral head (ONFH) in non-elderly patients versus senescence-driven ONFH in elderly patients, highlighting differences in etiology, pathology, imaging, and management.

Aspect	Non-elderly ONFH (typical)	Elderly ONFH (senescence-driven)
Common etiologies	Steroid use, alcohol abuse, trauma, hematologic disorders, idiopathic (1).	Often idiopathic/multifactorial; minor trauma or subchondral insufficiency fracture on osteopenic background (9, 70–72). Classic risk factors (steroids, alcohol) less frequently present, though comorbidities like diabetes or cardiovascular disease common (5, 21).
Age at onset	Typically, 20–50 years; rare in children, uncommon beyond 60 unless risk factors present (2, 3).	Late 60s onward; incidence rises in advanced age albeit under-recognized (4). Many patients in 70s–80s present with presumed OA that reveals ONFH/SIF on imaging (4).
Pathophysiological trigger	Often vascular insult: lipid emboli from steroid-induced hyperlipidemia causing capillary occlusion, or direct arterial injury in trauma (78). Osteocyte apoptosis prominent (especially steroid/alcohol ONFH) (17).	Often a mechanical/ischemic synergy: age-weakened bone succumbs to a subchondral microfracture which compromises blood flow, or age-impaired circulation leads to focal bone death under stress. Senescent cell accumulation and diminished remodeling capacity are central (4, 59). Osteocyte death more necrotic (passive) with less apoptotic signaling (17).
Demarcation and healing	Frequently shows reparative “creeping substitution”: sclerosis rim around necrotic zone on X-ray/MRI (49); incomplete but present revascularization at margins (29). Body may stabilize small lesions (some stage I lesions remain asymptomatic for years).	Poor demarcation: necrosis tends to diffuse into surrounding bone without strong sclerotic border (4). Reparative response weak—reflected by lack of border sclerosis and rapid progression. Healing attempts thwarted by senescent cell-driven inflammation and inadequate angiogenesis (33).
Rate of progression	Variable (17). Some cases (especially small or with removal of risk factor) progress slowly or not at all; others (large lesions, ongoing risk factors) progress to collapse in months. Without intervention, >70% of medium-large lesions will collapse within 2–3 years (156).	Generally fast progression. Many cases progress from symptom onset to collapse within weeks to few months (4). Rapid joint destruction (RDA-like) is more common (4). Unusual for elderly ONFH to remain asymptomatic/stable long once developed.
Bilateral involvement	Common in systemic etiologies (e.g., bilateral in >50% of steroid cases) (17). Idiopathic or trauma-related can be unilateral.	Often unilateral if due to local insufficiency fracture. Bilateral can occur if systemic cause (e.g., steroids) or if both hips have similar risk (e.g., severe osteoporosis), but less frequent than in younger steroid-induced ON (19).
Imaging – MRI	Early: Double-line sign on T2, homogeneous low signal band on T1 (smooth concave shape). BME may or may not be present depending on stage. Late: Crescent sign (fracture) on T1, collapse of articular surface, degenerative changes if chronic (18, 84, 85).	Early: Irregular subchondral low-signal line on T1 (often convex toward the articular surface), extensive BME common (48, 49). May resemble transient osteoporosis or insufficiency fracture. Progression: Rapid increase in BME and fracture line width; eventually articular collapse often involving much of head. Acetabular-side changes (edema, cysts) more often seen due to rapid degeneration (16).
Imaging – X-ray	Early: Normal or subtle sclerosis (79). Collapse: Crescent sign (subchondral lucent line) in anterolateral head; segmental flattening. Typically, a sequestrum of necrotic bone can be inferred, often with sclerotic border (2). Secondary OA changes if long-standing (osteophytes, moderate narrowing).	Early: May be normal or show slight osteopenia (79). After collapse: Femoral head flattening often diffuse (not just a segment). Joint space narrowing occurs early as collapse progresses (4). Sclerosis might be patchy or minimal. In severe cases, femoral head fragmentation and collapse leads to superior migration of the head (RDA pattern). Osteophytes are usually minimal because the timeline to arthrosis is short (4).
Histopathology	Necrotic zone with empty lacunae (dead osteocytes) and necrotic marrow fat (134). Border shows creeping substitution: osteoclasts resorbing dead bone, new woven bone forming (if ongoing repair). Osteogenic cells active at interface (unless risk factor ongoing) (135). Cartilage initially intact, later fissured once support lost.	Necrotic bone similar, but border zone often shows fibrous tissue and incomplete repair (157). Marrow infiltration by granulation tissue may be present but ineffective. Abundant repair tissue infiltration was noted early in elderly cases (4), yet this does not calcify into new bone effectively. Cartilage shows degeneration (fibrillation, loss) earlier in process, likely due to poor support and local inflammation. Possibly more subchondral cysts from marrow edema (157).
Cellular/molecular	High osteocyte apoptosis in steroid/alcohol ONFH (17); possibly less so in idiopathic. Focal increase in VEGF, bone morphogenetic proteins (BMPs) at repair front (135); systemic risk factors modulate these (steroids suppress osteogenic genes) (135). Lower baseline senescent cell burden in young.	Senescent cells prominent in lesion border, secreting SASP factors (IL-6, MMP-3, RANKL) that may accelerate damage (59). Downregulation of angiogenic/osteogenic genes relative to need (aging effect) (100). Systemic markers: often elevated inflammatory cytokines (inflammaging) (158), and DEXA might show low bone density. Some gene profiles (e.g., FOXO3, catalase) indicate poor oxidative stress handling (105).
Preferred treatments	Joint-preserving: Core decompression (often with bone graft or MSC augmentation for better success) for early stages (159); osteotomy in select cases (younger patients with specific lesion locations); tantalum rod or injection therapies experimental (136). If collapse and symptomatic: total hip arthroplasty (THA). Success of preservation higher if done early and patient biologically young (good healing capacity) (136).	Joint-preserving options are limited by poor bone stock and healing: Core decompression alone has high failure in elderly (bone may not heal the drilled tracts, risk of fracture) (159). CD with grafts or regenerative adjuncts attempted, but outcomes inferior to younger cohorts (138). Osteotomy usually not recommended (elderly rehab poorly, bone healing slow) (139). Emphasis on either augmenting mechanically (e.g., bone cement, prophylactic fixation for fracture) in early collapse or proceeding to THA (136). Many elderly patients end up with THA due to advanced presentation (137), but THA in very elderly has its own risks (though generally effective for pain relief) (5).
Outcome	With appropriate early treatment, young patients can often delay or avoid THA for many years; some small lesions even stabilize (139). However, without treatment, large lesions inevitably lead to end-stage arthritis (136). THA in younger patients has longevity concerns (will likely need revision in lifetime) (95).	Outcomes of joint-preservation are guarded; nearly half or more will progress to needing THA (137). Those managed non-operatively have persistent pain and disability if collapse occurs (136). THA outcomes in the elderly are generally positive in terms of pain relief, but perioperative risk must be managed (139). Elderly ONFH essentially often behaves like a rapidly progressive arthritic condition that, if the patient is fit, is definitively treated by arthroplasty.

As shown in Table 2, despite shared pathways involving femoral head necrosis and collapse, young and elderly ONFH differ fundamentally in etiology, progression, and host response. Young ONFH typically represents acute injury to healthy bone, while elderly ONFH reflects gradual failure of vulnerable bone under combined metabolic and mechanical stress. This distinction supports conceptually segregating “elderly osteonecrosis” in research and treatment paradigms, analogous to differentiating fragility from high-energy fractures. Current ONFH research predominantly pools all ages or focuses on younger patients, reflecting historical demographics. Geriatric-specific studies are urgently needed to address critical questions: Does core decompression benefit 75-year-old patients, or is primary arthroplasty more rational? Could anti-osteoporotic or anti-senescence therapies modify disease progression in elderly patients when unnecessary in younger cohorts? This age-stratified framework enables such targeted investigations.

### Proposed criteria for therapeutic stratification

Synthesizing the epidemiological and imaging evidence presented above, we propose establishing diagnostic boundaries to guide the therapeutic interventions discussed in the next section. Specifically, senescence-driven ONFH should be suspected when the following criteria converge: (1) Advanced Age (onset typically ≥60 years); (2) Absence of Major Risk Factors (no high-dose steroids or heavy alcohol use); (3) Atypical Imaging (irregular subchondral fractures or diffuse edema lacking the clear sclerotic rim of classic ONFH); and (4) Rapid Progression (structural collapse occurring faster than typical osteoarthritis). These boundaries are essential to restrict novel biological therapies (e.g., senolytics) to this specific phenotype, distinguishing it from classic osteonecrosis where standard protocols apply.

## Therapeutic implications: challenges and future directions in managing elderly ONFH

Understanding ONFH as a distinct entity in the elderly has direct implications for prevention and treatment in this growing population. The convergence of age-related pathogenic factors means traditional treatments may be less effective while opening doors to novel interventions targeting specific mechanisms (senolytics, anabolics, vasoprotectives).

### Why conventional treatments may fail or underperform in the elderly

Standard ONFH treatments aim to preserve the joint (early disease) or replace it (advanced disease). Joint-preserving procedures include core decompression, vascularized grafts, osteotomies, and regenerative therapies. However, several critical factors compromise their effectiveness in elderly patients.

Core decompression success relies on new bone formation to replace necrotic tissue and strengthen the area (136). In older patients, this reparative capacity is compromised. Biomechanical analysis confirms that the necrotic zone exhibits a 70% lower elastic modulus (*p* = 0.001) and significantly reduced bone volume fraction compared to proximal compressive trabeculae, rendering the bone structurally incompetent (127). These deficits translate into poor clinical survival: Yoon et al. reported a 48.4% conversion rate to total hip arthroplasty in elderly cohorts, identifying structural progression as a potent predictor of failure (Odds Ratio 11.8; 95% Confidence Interval 1.15–122.26) (137). Consequently, procedures effective in 35-year-olds might harm 75-year-olds whose bone cannot consolidate defects (138, 139).

Given these limitations, many orthopedic surgeons favor early total hip arthroplasty (THA) over head-salvage procedures in older ONFH patients. Modern THA demonstrates low perioperative mortality in optimized patients and provides excellent pain relief in ONFH cases (2). THA may represent the most definitive treatment for elderly patients with ONFH-related hip pain, transforming the condition into prosthesis management—which, given limited life expectancy, may not require revision if performed optimally (2). However, some elderly patients remain poor surgical candidates due to medical comorbidities, while costs and perioperative risks pose concerns in advanced age. Consequently, less invasive treatments that delay THA or serve as alternatives for non-surgical candidates remain necessary.

### Comprehensive management approach

An ideal management strategy for senescence-driven ONFH must be multidisciplinary and personalized, balancing established protocols with emerging biological concepts. The cornerstone of current care involves early identification and diligent risk factor modification. This includes tapering corticosteroids when feasible, managing lipids with statins (which may confer a vasculoprotective effect) (78), optimizing glycemic control in diabetics, and robustly treating osteoporosis. On this last point, bisphosphonates show particular promise; meta-analyses have demonstrated their potential to reduce femoral head collapse (140), and long-term follow-up has suggested a significant reduction in the need for surgery (141). For elderly patients already indicated for osteoporosis treatment, agents like bisphosphonates or denosumab may therefore provide the dual benefit of improving systemic bone density while potentially slowing local structural failure in the femoral head (74).

Beyond these established practices, the unique pathophysiology of senescence-driven ONFH invites the exploration of novel therapeutic hypotheses that target its core biological drivers. It must be underscored, however, that these emerging strategies are presently supported primarily by preclinical data or indirect clinical evidence, and their application to elderly ONFH remains largely conceptual, awaiting specific validation.

For instance, targeting cellular senescence represents a compelling, albeit investigational, frontier. The rationale is built on direct evidence that senescent cells accumulate at the necrotic border in human ONFH, secreting tissue-degrading factors (59). In parallel, preclinical work in aging mouse models has shown that senolytic drugs (e.g., dasatinib and quercetin) can rejuvenate bone formation (142, 143). This makes it a logical hypothesis that senolytics, or senomorphics that modulate the inflammatory secretory phenotype (144), could disrupt the local pathological cascade. However, this remains an extrapolation from fundamental biology and animal studies. Bridging this gap requires navigating currently unmapped pharmacodynamic and toxicologic intermediates. Crucially, to date, no preclinical studies have validated the efficacy or delivery of these agents specifically within the compromised vascularity and unique hypoxic, lipid-loaded microenvironment of the necrotic femoral head, which may significantly alter drug bioavailability. Notably, the systemic administration of senolytic cocktails (e.g., dasatinib) carries established risks of hematologic and hepatic toxicity (145) and poses critical concerns regarding drug–drug interactions (e.g., with anticoagulants or statins) in this typically polymedicated population. Consequently, while general side effects are known, the specific safety profile and benefit–risk ratio of such agents in a frail, elderly population with ONFH remain undefined, necessitating rigorous preclinical safety studies and subsequent, ONFH-specific clinical trials.

Similarly, strategies aimed at enhancing the body’s limited endogenous repair capacity offer another hypothetical avenue. Anabolic agents like teriparatide (PTH 1–34) stimulate bone formation and may indirectly promote angiogenesis (146), but evidence in ONFH is limited to preliminary studies and case series that require substantiation. Other concepts, such as local growth factor delivery (VEGF, BMPs) or the use of vasculoprotective agents like pentoxifylline and tocopherol, are extrapolations from their promise in animal models (147) or their use in other ischemic conditions like osteoradionecrosis (148, 149). While theoretically attractive, their efficacy and safety within the complex, hypoxic, and inflammatory milieu of an aging, necrotic femoral head are uncertain.

While these biological interventions are being investigated, surgical management remains the pragmatic reality. For elderly patients diagnosed at pre-collapse stages who are reasonable surgical candidates, combining core decompression with mechanical augmentation (e.g., bone grafts, cements, or porous tantalum rods) is a conceptually appealing approach to address the underlying biomechanical weakness (139, 150, 151). However, for many, THA will likely be the eventual solution. In this context, surgical decision-making must prioritize the patient’s functional status and frailty index over purely radiographic or biological parameters. Given that arthroplasty yields excellent and durable results in older patients with modest activity demands, the threshold to proceed to THA is justifiably lower than in younger cohorts, especially when early collapse threatens to damage the acetabulum and complicate future surgery (152, 153).

Finally, prevention remains paramount, requiring proactive management of bone health, vascular risk factors, and polypharmacy, alongside consideration of prophylactic therapies for high-risk individuals (78). A coordinated, geriatric-focused approach is essential, integrating orthopedic diagnosis with endocrinological and geriatric expertise to treat not just the joint, but the underlying aging biology.

## Limitations

**Figure 4:**
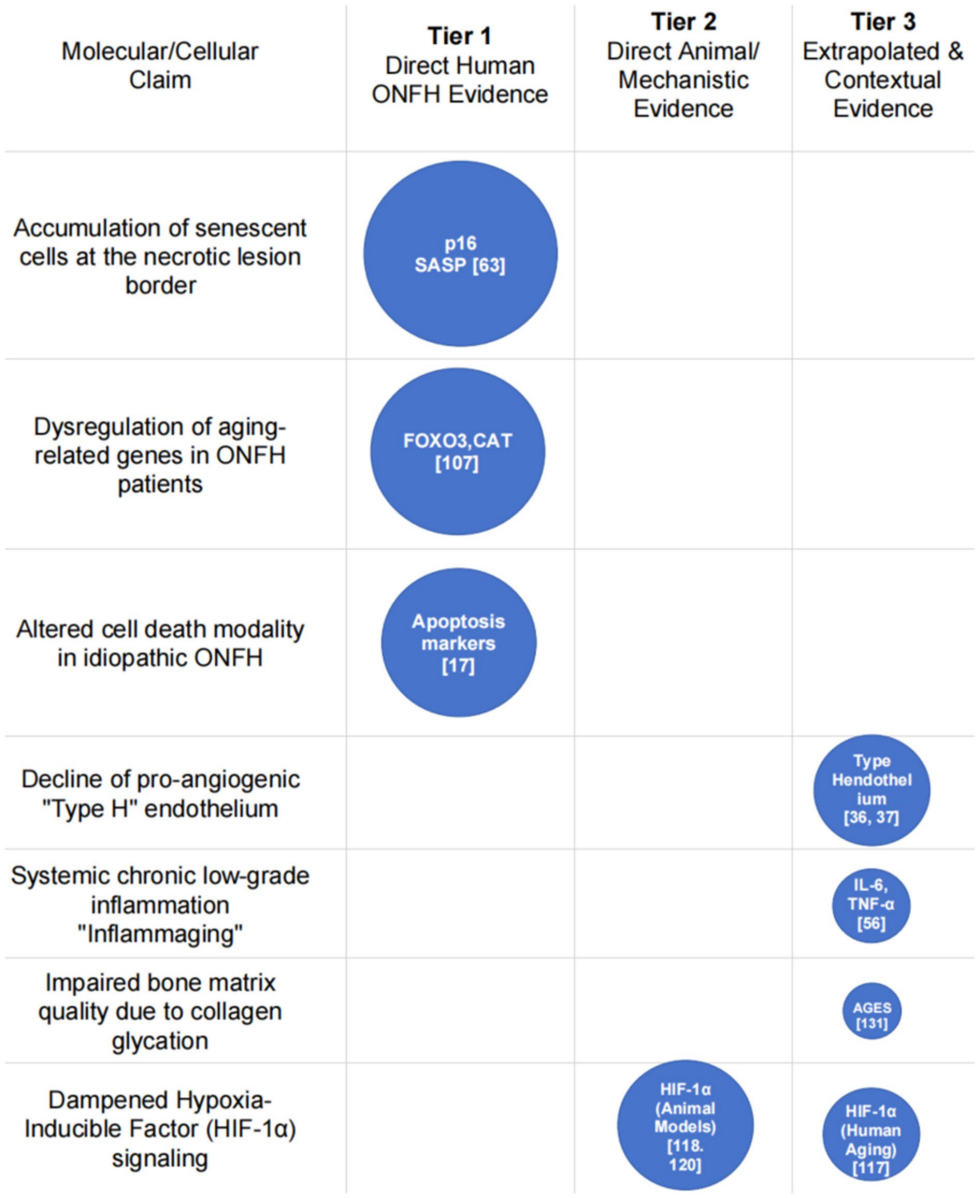
Molecular evidence map for the pathogenesis of senescence-driven ONFH.

We acknowledge that a primary limitation of this work is its nature as a conceptual framework, constructed by synthesizing evidence from parallel fields. Currently, no validated diagnostic definition or specific molecular biomarker exists to strictly differentiate this entity from osteoporotic subchondral insufficiency fractures, reflecting the significant pathophysiological overlap in the geriatric population. This approach is necessitated by the current scarcity of molecular studies focused specifically on idiopathic ONFH in the elderly. Specifically, no unified dataset currently links molecular, imaging, and biomechanical parameters within the same patient cohort; thus, our proposed integration remains a theoretical synthesis. We also explicitly acknowledge that due to the paucity of ONFH-specific trials in the elderly, a significant portion of the supporting molecular evidence is extrapolated from broader skeletal aging research. Additionally, the variability in age thresholds across the cited literature (ranging from >60 to >75 years) introduces unavoidable heterogeneity into the synthesized clinical phenotypes. To provide maximum clarity on this point and to transparently delineate the origins of our key molecular arguments, we have developed a “Molecular Evidence Map” (Figure 4). This figure visually maps each molecular claim to its evidence source, using circle size to represent the evidence weight, thereby making the evidence hierarchy immediately interpretable.

To clarify this distinction for the reader: statements regarding the presence of senescent cells (p16INK4a, p21) and a senescence-associated secretory phenotype (SASP) at the necrotic border are directly supported by data from human ONFH tissue (59). Likewise, discussions on aging-related gene profiles (e.g., FOXO3, CAT) (105) and differing cell death modalities (17) are based on studies of ONFH patient samples. However, these cohorts were not exclusively elderly or idiopathic. In contrast, other key concepts are well-supported extrapolations: the roles of systemic “inflammaging” (52), the decline of pro-angiogenic “type H” endothelium (33), and biomechanical failure from advanced glycation end-products (AGEs) are inferred from established principles in gerontology and research into SIF and osteoporosis (47, 128).

This evidence gap underscores the urgent need for future research to directly validate our proposed mechanisms. Specifically, comparative omics analyses of femoral head tissue from young versus elderly idiopathic ONFH cohorts, alongside the development of relevant geriatric animal models, are critical next steps.

## Conclusion

The recognition of osteonecrosis of the femoral head in elderly patients as a distinct pathophysiological entity carries important implications for science and clinical practice, as senescence-driven ONFH represents not merely “regular osteonecrosis in an older person,” but rather the outcome of unique age-related susceptibilities—an unstable fusion of fragile vasculature, fatty weak bone, and chronic inflammation culminating in femoral head failure where the elderly femoral head exists on a narrower margin of safety with less adaptable blood supply, less robust bony framework, and often blunted reparative machinery. Evidence from epidemiology, imaging, histology, and molecular studies reveals aging introduces qualitative differences (disease intertwined with senescence marked by cellular aging and mechanical insufficiency) and quantitative differences (faster progression and poorer outcomes) in ONFH, underscoring the need for a paradigm shift toward maintaining high suspicion in older patients, earlier MRI use, age-stratified clinical trials, and expanding beyond traditional orthopedic approaches to explore geroprotective interventions like senolytics, anabolics, or anti-resorptives. The future may lie in hybrid approaches combining mechanical stabilization (cement injection, implants) with biological repair stimulation (teriparatide, anti-senescent agents), as evidence shows statins’ protective effects, bisphosphonates preventing collapse, and MSC secretome reducing collapse in animal models hint that metabolic and cellular interventions can alter ONFH course in the elderly.

Framing ONFH in the elderly as a geriatric condition exemplifies how age modifies disease not just in degree but in kind, enabling new frameworks for diagnosis (identifying what was missed as “just arthritis”), prevention (prophylactic bone-preserving agents for high-risk patients), and treatment (tailored interventions maintaining mobility and independence rather than one-size-fits-all protocols), ultimately challenging us to integrate geriatric medicine insights with orthopedic practice to treat not just the disease but the aging biology underpinning it, potentially transforming an almost inevitable march to collapse and arthroplasty into a manageable aspect of aging.

## Data Availability

The original contributions presented in the study are included in the article/supplementary material, further inquiries can be directed to the corresponding authors.
